# N-nitroso-N-ethylurea activates DNA damage surveillance pathways and induces transformation in mammalian cells

**DOI:** 10.1186/1471-2407-14-287

**Published:** 2014-04-24

**Authors:** Satish Bodakuntla, Libi Anandi V, Surojit Sural, Prasad Trivedi, Mayurika Lahiri

**Affiliations:** 1 Indian Institute of Science Education and Research, Pune, Maharashtra 411008, India; 2 Current address: Department of Molecular and Integrative Physiology, University of Michigan, Anne Arbor, MI 48109, USA; 3 Current address: Department of Biochemistry & Molecular Genetics, University of Virginia Medical Center, Charlottesville, VA 22908, USA

**Keywords:** N-nitroso-N-ethylurea, DNA lesions, Epithelial - mesenchymal transition, Mismatch repair, *O*^*6*^-ethylguanine, DNA damage response, Checkpoints, Cell cycle, Comet assay, 3-dimesional cultures, Transformation

## Abstract

**Background:**

The DNA damage checkpoint signalling cascade sense damaged DNA and coordinates cell cycle arrest, DNA repair, and/or apoptosis. However, it is still not well understood how the signalling system differentiates between different kinds of DNA damage*.* N-nitroso-N-ethylurea (NEU), a DNA ethylating agent induces both transversions and transition mutations.

**Methods:**

Immunoblot and comet assays were performed to detect DNA breaks and activation of the canonical checkpoint signalling kinases following NEU damage upto 2 hours. To investigate whether mismatch repair played a role in checkpoint activation, knock-down studies were performed while flow cytometry analysis was done to understand whether the activation of the checkpoint kinases was cell cycle phase specific. Finally, breast epithelial cells were grown as 3-dimensional spheroid cultures to study whether NEU can induce upregulation of vimentin as well as disrupt cell polarity of the breast acini, thus causing transformation of epithelial cells in culture.

**Results:**

We report a novel finding that NEU causes activation of major checkpoint signalling kinases, Chk1 and Chk2. This activation is temporally controlled with Chk2 activation preceding Chk1 phosphorylation, and absence of cross talk between the two parallel signalling pathways, ATM and ATR. Damage caused by NEU leads to the temporal formation of both double strand and single strand breaks. Activation of checkpoints following NEU damage is cell cycle phase dependent wherein Chk2 is primarily activated during G2-M phase whilst in S phase, there is immediate Chk1 phosphorylation and delayed Chk2 response. Surprisingly, the mismatch repair system does not play a role in checkpoint activation, at doses and duration of NEU used in the experiments. Interestingly, NEU caused disruption of the well-formed polarised spheroid archithecture and upregulation of vimentin in three-dimensional breast acini cultures of non-malignant breast epithelial cells upon NEU treatment indicating NEU to have the potential to cause early transformation in the cells.

**Conclusion:**

NEU causes damage in mammalian cells in the form of double strand and single strand breaks that temporally activate the major checkpoint signalling kinases without the occurrence of cross-talk between the pathways. NEU also appear to cause transformation in three-dimensional spheroid cultures.

## Background

Alkylating agents are a structurally diverse group of DNA damaging compounds which form adducts at ring nitrogen (N) and extracyclic oxygen (O) atoms of DNA bases [1]. N-nitroso-N-ethylurea (NEU), a simple monofunctional S_N_1 type-DNA ethylating agent, forms the modified base *O*^*6*^-ethylguanine (*O*^*6*^EtG) which mispairs with thymine during DNA replication and thus primarily induces A:T to T:A transversions or A:T to G:C transition mutations [2,3]. NEU has been traditionally characterised as a severely potent transplacental teratogen and carcinogen in rodents [4,5]. In vertebrates, the mismatch repair proteins, namely Msh2-Msh6 and Mlh1-Pms2 heterodimers, play a pivotal role in mediating the mutagenic and cytotoxic effects of *O*^*6*^EtG lesions [6,7]; however the mechanisms are still controversial. According to one model, futile cycles of mismatch repair-induced excision and repair of erroneously paired thymine nucleotides opposite *O*^*6*^EtG lesions cause formation of recurring single strand breaks (SSBs). These gaps in the genome and double strand breaks (DSBs) that form at these sites during the next replication cycle have been proposed to be mediating the cytotoxic effects of different alkylating agents [8]. However according to an alternate model, recognition of *O*^*6*^EtG:T mispairs by the mismatch repair proteins can directly recruit DNA damage response kinases at the site of DNA damage which possibly elicits cell cycle checkpoint activation and apoptosis [9]. Interestingly, at high doses, cytotoxicity of S_N_1 type-alkylating agents has been shown to be largely mismatch repair independent [10]. Hence the cellular pathways that collectively modulate sensitivity to DNA alkylation damage involve direct crosstalk, overlap in substrates and recruitment of alternative pathways for processing of intermediates.

The pathways that sense damaged DNA and coordinate DNA repair, cell cycle arrest and/or apoptosis comprise the DNA damage checkpoint signalling cascade. The sensor or apical kinases that detect the damaged DNA belong to the phosphoinositide 3-kinase related kinase (PIKK) family. These kinases, namely ATM (ataxia-telangiectasia mutated) and ATR (ATM and Rad3-related), initiate a cascade of phosphorylation events which mediate cell cycle arrest, DNA repair and apoptosis [11]. The increased local concentration of ATM at the DSB sites is important to boost phosphorylation of ATM targets, including signal mediators such as the Chk2 kinase [12]. ATR responds primarily to stalled replication forks, base adducts and DNA cross-links, and relays the signal by phosphorylating Chk1 kinase and a large subset of ATM substrates [13]. However this paradigm of ATM and ATR signalling through two independent and alternate pathways was recently challenged and redefined by several reports showing that ATR can be activated directly in response to DSBs specifically in S and G2 phases of the cell cycle [14,15]. Recruitment of ATR to ionising radiation (IR)-induced DSBs occurs in an ATM and Mre11-Rad50-Nbs1 (MRN) complex-dependent manner at time points following ATM activation [16]. Though DNA alkylating agents do not directly induce strand breaks, low doses of S_N_1 type-methylating agents have been shown to induce activation of the apical kinases, ATM and ATR, and their downstream substrates [10,17]. Most studies suggest that checkpoint activation can occur only in the second G2 phase after DNA alkylation damage, however few findings have reported ATM activation within 3 hrs of treatment with a prototypical S_N_1 type-methylating agent [18]. Furthermore, SSBs have been shown to accumulate as primary lesions in cells after 2 hrs of NEU damage [19]. These findings, being contrary to the standard model of DNA alkylation damage, have led to the possibility that S_N_1 type-alkylating agents can induce strand breaks in a replication-independent manner.

Loss of cellular architechture and polarity of breast tissue is one of the early markers for onset of breast cancer. This loss in cellular morphology can be phenocopied using three-dimensional (3D) cultures of human mammary epithelial cells, MCF10A. MCF10A are immortalised, non-transformed human mammary epithelial cells when grown in 3D matrices, exhibit a number of features of normal breast epithelium [20]. MCF10A cells form multicellular acini-like spheroids which represent the layer of basal epithelial cells surrounding a hollow lumen in the lobule of human mammary gland [21]. The morphology of these acini are disrupted in malignancy, such as an increase in size and elongation of acini [22]. As transformation progresses, the acini lose their polarisation and some may even form multi-acinar structures [21]. Another characteristic of transformed cells is their ability to invade and metastasise to the other tissues. During the process of transformation the epithelial cells are said to undergo ‘epithelial - mesenchymal’ (EMT) transition [23-25].

In this study, we have investigated the activation of DNA damage response kinases in human cancer cell lines following 2 hours treatment with different doses of NEU. Mismatch repair-proficient and mismatch repair-deficient cells were used to address the dependence on an active mismatch repair system for signalling to apical kinases of the DNA damage response signalling cascade after ethylation damage. We also explored the possibility of crosstalk and/or interdependence between the two canonical DNA damage response pathways, namely ATM through Chk2 and ATR through Chk1, post-NEU treatment for 2 hours. The data indicate presence of a mismatch repair-independent and cell cycle phase-dependent mechanism of checkpoint activation in mammalian cells immediately after treatment with a prototypical S_N_1 type-ethylating agent. Using the 3D platform to investigate whether NEU has the potential to cause transformation of breast epithelial cells grown as spheroids, it was observed that upon NEU treatment to MCF10A acinar cultures, the well organised polarised structures were completely distrupted upon transformation. Vimentin, an EMT marker was also observed in the NEU-treated breast acini, thereby indicating NEU to cause an EMT-like phenotype in the transformed breast epithelial cells grown in 3D.

## Methods

### Cell lines and culture conditions

MCF7 cell line was purchased from European Collection of Cell Cultures (ECACC). HeLa and HCT 116 cell lines were generous gifts from Dr. Sorab Dalal (ACTREC, Mumbai, India). DLD1 cell line was a kind gift from Dr. Thomas Ried (NCI, NIH, USA). MCF10A cell line was a generous gift from Prof. Raymond C. Stevens (The Scripps Research Institute, California, USA). All cell lines were grown in High Glucose Dulbecco’s Modified Eagle Medium (DMEM; Invitrogen or Lonza) containing 10% fetal bovine serum (FBS; Invitrogen), 2 mM L-glutamine (Invitrogen) and 100 units/mL penicillin-streptomycin (Invitrogen). MCF10A cells were grown in High Glucose DMEM without sodium pyruvate (Invitrogen) containing 5% horse serum (Invitrogen), 20 ng/mL EGF (Sigma), 0.5 μg/mL hydrocortisone (Sigma), 100 ng/mL cholera toxin (Sigma), 10 μg/mL insulin (Sigma) and 100 units/mL penicillin-streptomycin (Invitrogen) and were resuspended during sub-culturing in High Glucose DMEM without sodium pyruvate containing 20% horse serum and 100 units/mL penicillin-streptomycin (Invitrogen). Cells were maintained in 100 mm tissue-culture treated dishes (Corning) at 37°C in humidified 5% CO_2_ incubator (Thermo Scientific).

### Chemicals and antibodies

Dimethyl sulfoxide (DMSO), N-nitroso-N-ethylurea (NEU), neocarzinostatin (NCS), thiazolyl blue tetrazolium bromide (MTT), thymidine, nocodazole, RNase A and propidium iodide (PI) were purchased from Sigma-Aldrich. Selective ATM inhibitor KU 55933 and DNA-PK inhibitor DMNB were obtained from Tocris Bioscience. VE 821, a potent and selective ATR kinase inhibitor was purchased from Axon Medichem. Monoclonal antibodies for Chk1 and Msh2 were bought from Santa Cruz Biotechnology. Polyclonal antibodies for phospho-Chk1 (Ser345), phospho-Chk2 (Thr68) and monoclonal antibodies for Chk2 and RPA32 were purchased from Cell Signaling Technology. Polyclonal antibody for phospho-RPA (Thr21) and monoclonal antibodies for phospho-ATM (Ser1981) and ATM were obtained from Abcam. Monoclonal antibodies for γH2AX (Ser139) and α6 integrin were bought from Millipore while α-tubulin was from Sigma. Monoclonal antibodies for vimentin, E-cadherin and β-catenin were purchased from Abcam. Peroxidase-conjugated AffiniPure goat anti-mouse, anti-rabbit and anti-rat IgG (H + L) as well as AffiniPure F(ab’)_2_ fragment goat anti-mouse IgG, F(ab’)_2_ fragment specific were obtained from Jackson Immuno Research. 4′, 6-Diamidino-2-phenylindole dihydrochloride (DAPI), Alexa Fluor 488 donkey anti-rabbit IgG (H + L) and Alexa Fluor 568 goat anti-mouse IgG (H + L) were bought from Invitrogen.

### MTT-based cytotoxicity assay

Cells were seeded at a density of 10^4^ cells per well in 96-well flat bottom tissue culture treated plates (Corning) and maintained at 37°C for 16 hours. Cells were then treated with NEU for 2 hours. Medium containing drug was aspirated and fresh growth medium containing 0.5 mg/mL MTT was added to cells. Plates were maintained in dark at 37°C for 4 hours. Medium-MTT mixture was aspirated and MTT-formazan crystals were dissolved in DMSO. Plates were kept on a nutating shaker at room temperature (RT) for 5 minutes and absorbance was recorded at 570 nm using a Varioskan Flash Multimode Plate Reader (Thermo Scientific).

### Drug treatment and time course assays

Cells were seeded at a density of 10^6^ cells per well in 6-well tissue culture treated plates (Corning) and maintained at 37°C for 16 hours. Cells were then treated with NEU by direct addition of drug to the culture medium for 2 hours (unless otherwise indicated). Control cells were treated with equivalent volume of DMSO (drug solvent). For ATM and DNA-PK inhibition, cells were treated with 10 μM KU 55933 and 25 μM DMNB, respectively, immediately prior to addition of drug while for ATR inhibition 10 μM VE 821 was added one hour prior to addition of NEU to the cells. For time course studies, cells were treated with NEU for different time periods ranging from 0 to 120 minutes. After drug treatment, medium containing NEU was aspirated and cells were washed once with 1X phosphate buffered saline (PBS; PAN-Biotech GmbH). Cells were lysed in sample buffer containing 0.06 mM Tris (pH 6.8), 6% glycerol, 2% sodium dodecyl sulphate (SDS), 0.1 M dithiothreitol (DTT) and 0.006% bromophenol blue and lysates were stored at - 40°C.

### Single cell gel electrophoresis (Comet assay)

DNA strand breaks were detected using single cell gel electrophoresis/comet assay, using standard protocols [26]. Comet slides were then stained with ethidium bromide at a concentration of 2 μg/ml for 5 minutes and then were scored for comets immediately. Images were acquired using epiflourescence microscope at 20X magnification. Randomly selected 50 cells were analysed per sample. Amount of DNA SSBs and DSBs were measured and represented as length of tail and relative DNA content in tail.

### siRNA knockdown

siRNA duplexes targeting Msh2, Msh6 and LacZ were purchased from Dharmacon (Thermo Scientific). Sense sequences of the siRNA are: Msh2, 5′-ACAGAAUAGAGGAGAGAUUUU-3′; Msh6, 5′-GAAUACGAGUUGAAAUCUAdTdT-3′; LacZ, 5′-CGUACGCGGAAUACUUCGAdTdT-3′. HeLa cells were seeded at a density of 0.3 X 10^5^ cells per well in 12-well tissue culture treated plates (Corning) and maintained at 37°C for 24 hours. Transfections were performed with a final siRNA concentration of 100 nM using X-tremeGENE siRNA transfection reagent (Roche) diluted in OptiMEM I Reduced Serum Medium (Invitrogen). DMEM supplemented with 30% FBS was added 4 hours post-transfection to achieve a final FBS concentration of 10% in the wells. After 24 hours, siRNA transfection was repeated for each set. Cells were maintained at 37°C for an additional 48 hours and NEU damage was induced before lysis using same procedure as described earlier.

### Immunoblot analysis

Cell lysates were resolved using sodium dodecyl sulphate polyacrylamide gel electrophoresis (SDS-PAGE) and transferred to Immobilon-P polyvinylidene difluoride (PVDF) membrane (Millipore). Blocking was performed in 5% (w/v) skimmed milk (SACO Foods, USA) for non-phospho antibodies or 4% (w/v) Block Ace (AbD Serotec) for phospho-specific antibodies prepared in 1X tris buffered saline containing 0.1% Tween 20 (1X TBS-T) for 1 hour at RT. Blots were incubated for 3 hours at RT (or for 16 hours at 4°C) in primary antibody solution. Following washes, blots were incubated with peroxidase-conjugated secondary antibody solution prepared in 5% (w/v) skimmed milk in 1X TBS-T for 1 hour at RT following which blots were developed using Immobilon Western Detection Reagent kit (Millipore) and visualised using ImageQuant LAS 4000 (GE Healthcare). All western data were quantified using minimum three independent experiments and have been denoted as fold-difference over respective controls for each blot.

### Cell cycle synchronisation

Cell cycle synchronisation for S (double thymidine block) and G2 (thymidine-nocodazole block) phases were performed following the protocol mentioned by Whitfield et al. [27]. For the S phase synchronisation, HeLa cells were seeded at a density of 10^5^ cells per well in 6-well tissue-culture treated plates while for synchronisation in G2 phase, cells were seeded at 2.5 X 10^5^ cells per well in 6-well tissue-culture treated plates. The cells were released into DMEM containing 10 mM NEU and harvested at different time points.

### Cell cycle analysis

HeLa cells were synchronised as mentioned above and harvested by trypsinisation. Cells were washed twice with 1X PBS and fixed with 70% ethanol at least overnight at 4°C. Cells were then washed twice with 1X PBS, resuspended in a solution containing 20 mg/ml RNase A and 1 mg/ml PI and incubated at 37°C for 1 hr. Cell cycle analysis was performed using FACSCalibur flow cytometer (BD Biosciences) and data was analysed using ModFit (Verity Software House, Topsham, ME, USA).

### Immunofluorescence analysis

Cells were seeded at a density of 2 X 10^5^ cells per well on top of glass cover slips (Micro-Aid, India). Following drug treatment, cells were fixed using 4% formalin (Macron Chemicals) and were permeabilised using 0.5% Triton-X-100 for 5 minutes at RT. Cells were blocked with 10% (v/v) goat serum (Abcam), stained with primary antibody and then incubated with secondary antibody. For FITC-conjugated γH2AX (Ser139), the secondary antibody step was skipped. Cells were then counterstained with 0.5 μg/ml DAPI and mounted on glass slides (Micro-Aid, India). Slides were visualised under an Axio Imager.Z1 ApoTome microscope or a LSM 710 laser scanning confocal microscope (Carl Zeiss, GmbH). All microscopy images, unless otherwise specified, were captured using 63X oil-immersion objective.

### 3D “on-top” culture

The 3D on top cultures were set up in 8-well chamber coverglass (Nunc Lab tek, Thermo Scientific) using protocol described previously [21,28]. Cells were seeded at a density of 0.5 X 10^4^ cells per well. Cultures were maintained for 20 days and medium was supplemented every 4 days [21]. For drug treatments, NEU was directly added to the culture medium on day 0 and 2. (Day 0 being the day of seeding).

### In-well 3D culture extraction and immunofluorescence

The acini were fixed on the 20^th^ day using 4% paraformaldehyde (PFA) (freshly prepared in PBS, pH 7.4), permeabilised using PBS containing 0.5% Triton-X-100 for 10 minutes at 4°C, and immunostaining was done using standard protocols [21,28]. 3D structures were visualised under a Zeiss LSM 710 laser scanning confocal microscope (Zeiss, GmbH). All immunofluorescence images, unless otherwise specified, were captured using 63X oil-immersion objective.

### Statistical analysis

Data represented in comet assay graphs are mean +/− standard error of parameters recorded from three independent experiments. Student’s t-test was used to analyse the statistical significance of fold-difference between treated and control samples in the western blots. Student’s t-test was also used to analyse the statistical significance of difference in tail length. The results for % DNA in tail were analysed using nonparametric test one-tailed Mann Whitney U test. One way ANOVA was used to analyse the statistical significance of difference in the relative DNA content in tail for time course experiments. The results for % DNA in tail were also confirmed using nonparametric tests (Kruskal-Wallis test). The data was analysed using GraphPad Prism software (GraphPad Software, La Jolla, CA, USA), and p < 0.05 has been considered as significantly different.

## Results

### NEU damage activates DNA damage response kinases

**Figure 1:**
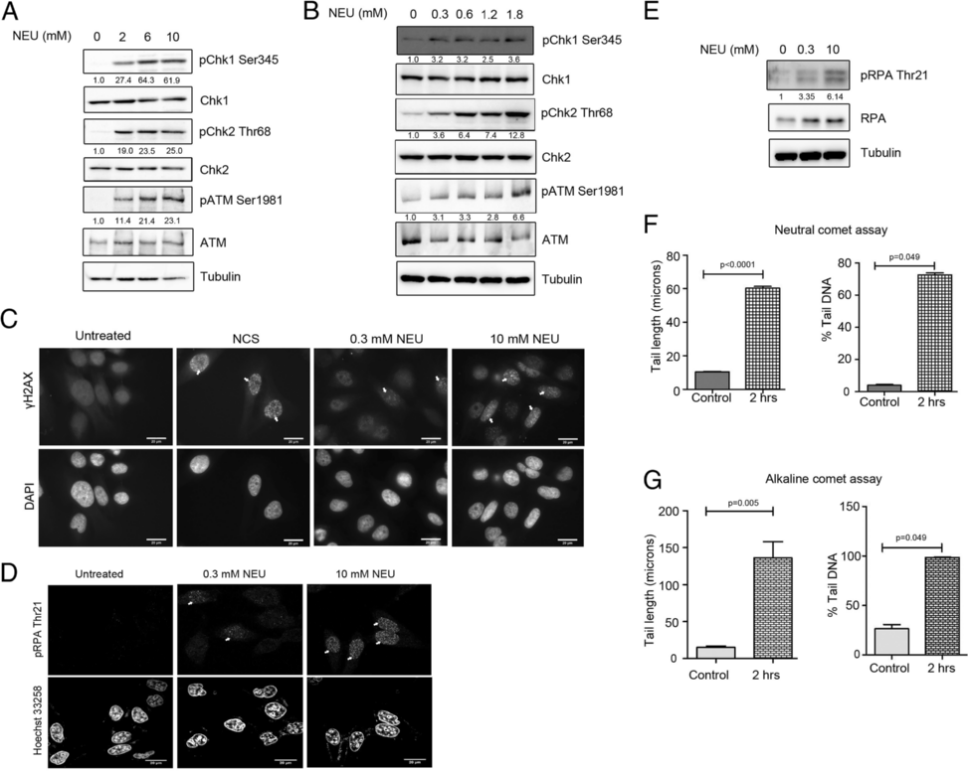
**NEU causes activation of checkpoint signalling pathways in a dose dependent manner. (A)** MCF7 cells were treated with 0, 2, 6 and 10 mM NEU for 2 hours and lysates were analysed for activation of checkpoint proteins by immunoblotting. **(B)** MCF7 cells were treated with 0, 0.3, 0.6, 1.2 and 1.8 mM NEU for 2 hours and lysates were analysed for activation of checkpoint proteins by immunoblotting. **(C)** HeLa cells were treated with 0.3 and 10 mM NEU for 1 hour, fixed and analysed for γH2AX foci formation by immunostaining. DMSO was used as negative control and neocarzinostatin (NCS), an IR-mimetic drug, was used as positive control at a concentration of 200 ng/ml. Scale bar: 20 μM. **(D)** HeLa cells were treated with 0.3 and 10 mM NEU for 2 hours, fixed and analysed for pRPA foci formation by immunostaining. DMSO was used as negative control. Scale bar: 20 μM. **(E)** HeLa cells were treated with 0, 0.3 and 10 mM NEU for 2 hours and lysates were analysed for phosphorylation of RPA. **(F and G)** MCF7 cells treated with 0 and 10 mM NEU for 2 hours were collected, embedded in agarose and layered on slides. Cells were subjected to lysis followed by electrophoresis in neutral and alkaline conditions respectively. N = 150 cells (50 cells/experiment). Data shown are mean +/− standard error. The results for % DNA in tail and tail length are significantly different at p < 0.05 in Mann Whitney U test and student’s t test respectively.

To evaluate the cytotoxicity induced by NEU in MCF7 (breast adenocarcinoma origin) and HeLa (cervical adenocarcinoma origin) cell lines, MTT-based cell viability assay was used (see Additional file 1: Figure S1, A and B respectively). In the dose range at which cell viability was between 50% and 100%, NEU induced phosphorylation of the DNA damage response kinases ATM, Chk2 and Chk1 was observed in MCF7 and HeLa cells in a dose dependent manner (Figure 1A and Additional file 1: Figure S1C). To investigate whether DNA damage cascades are activated on exposure to NEU doses at which cell viability is higher than 70%, we treated MCF7 and HeLa cells with drug concentrations lower than 2 mM. Interestingly, phosphorylation of ATM, Chk2 and Chk1 were also detected in the low dose range of NEU (Figure 1B and Additional file 1: Figure S1D). Since both DSB and SSB response pathways were activated after exposure to NEU, we sought to visualise the presence of these breaks immediately after NEU damage. γH2AX foci formation was observed in HeLa and MCF7 cells after 1 hour of NEU damage and these nuclear foci intensified with increase in concentration of the drug (Figure 1C and Additional file 1: Figure S1E). NEU damage also led to phosphorylation of RPA at threonine 21 residue and induced localisation of phospho-RPA proteins to nuclear foci in HeLa cells within 2 hour of addition of the drug (Figure 1D and E). Neutral and alkaline comet assays were performed to further confirm the formation of DSBs and SSBs respectively. A significant increase in comet formation was observed in MCF7 cells post NEU damage for 2 hours (Figure 1F and G; Additional file 2: Figure S2A and B) compared to control cells. Together these data suggests that NEU induces formation of both SSBs and DSBs within two hours, which leads to the activation of the DNA damage response signalling cascades, namely Chk1 and Chk2 with formation of damage-induced foci.

### NEU-induced DNA damage response activation is independent of the mismatch repair system

**Figure 2:**
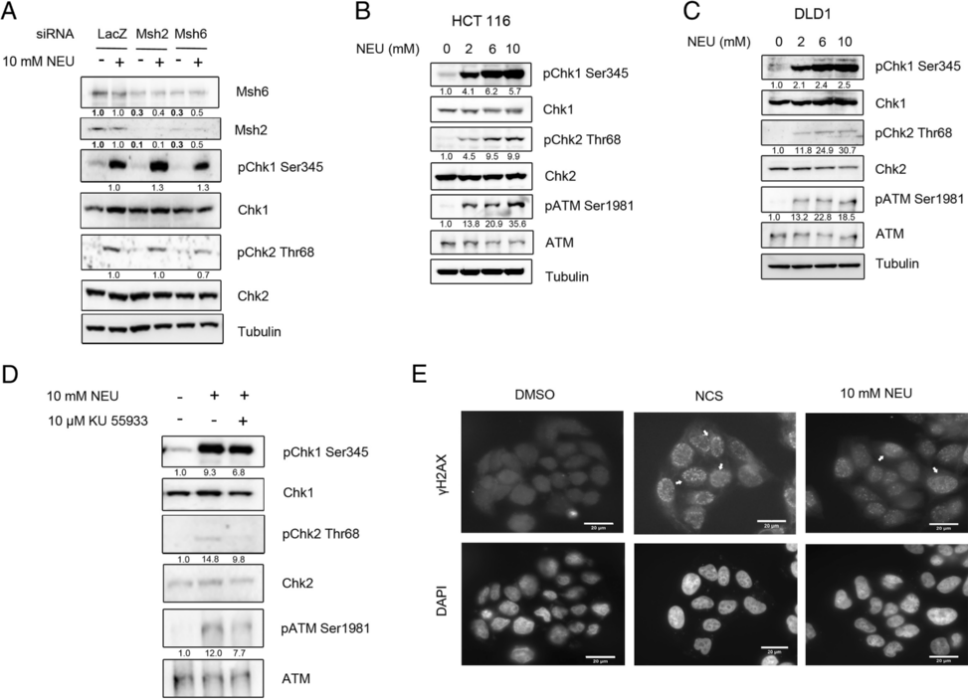
**Checkpoint activation in response to NEU-induced damage is independent of mismatch repair. (A)** siLacZ, siMsh2 and siMsh6 transfected HeLa cells were treated with 10 mM NEU for 2 hours and lysates were analysed for activation of checkpoint proteins by immunoblotting. **(B)** HCT116, a MLH1-deficient cell line, was treated with 0, 2, 6 and 10 mM NEU for 2 hours and lysates were analysed for activation of checkpoint proteins by immunoblotting. **(C)** DLD1, a MSH6 deficient cell line, was treated with 0, 2, 6 and 10 mM NEU for 2 hours and lysates were analysed for activation of checkpoint proteins by immunoblotting. **(D)** DLD1 cells were treated with 10 mM NEU in presence of 10 μM KU 55933 (ATM inhibitor) for 2 hours and lysates were analysed for activation of checkpoint proteins by immunoblotting. **(E)** DLD1 cells were treated with 10 mM NEU for 1 hour, fixed and analysed for formation of γH2AX foci by immunostaining. DMSO was used as negative control and 200 ng/ml NCS, an IR-mimetic drug, was used as positive control. Scale bar: 20 μM.

Earlier reports have shown mismatch repair proteins to mediate checkpoint activation and downstream cytotoxic effects induced upon exposure to DNA alkylating agents [10,17,29,30]. To investigate whether activation of DNA damage response after NEU damage was dependent on mismatch repair, we performed individual knockdowns of the mammalian MutS homologs, Msh2 and Msh6, in HeLa cells. Knocking down of Msh2 or Msh6 using siRNAs against the endogenous proteins did not affect the checkpoint response following 2 hours of NEU treatment (Figure 2A). Phosphorylation of Chk1 at serine 345 and of Chk2 at threonine 68 was observed in Msh2 or Msh6 knocked down cells following NEU damage. The levels of phospho-Chk1 (Ser 345) in NEU-treated Msh2 or Msh6 RNAi-depleted cells were simlar to that of LacZ RNAi knocked down cells in the presence of NEU (1.3 fold for Msh2 and Msh6 siRNA lanes compared to LacZ control in the presence of damage). Similarly the levels of Chk2 phosphorylation in Msh2 or Msh6 RNAi-depleted cells following NEU damage were 1 and 0.7 fold difference in comparison to NEU damaged LacZ lane and hence the activation of both Chk1 and Chk2 remain unperturbed in NEU-treated Msh2 or Msh6 RNAi-depleted cells. To further explore DNA damage response activation after NEU damage mismatch repair-deficient cell lines, HCT 116, a MLH1 deficient cell line of colon cancer origin and DLD1, a MSH6 deficient (frame-shift mutation in Msh6) cell line derived from colorectal adenocarcinoma were used in the experiments. In HCT 116, activation of the DNA damage response kinases ATM, Chk2 and Chk1 were observed after exposure to NEU concentrations from 2 mM to 10 mM for 2 hours (Figure 2B). Similar results were obtained for DLD1 cells (Figure 2C). Though it has been previously reported that the DLD1 cell line is Chk2 deficient [31], we could detect low amounts of phosphorylation of Chk2 at threonine 68 position in DLD1 cells which was reduced in presence of an ATM autophosphorylation inhibitor, KU 55933 (Figure 2D). To confirm that DNA damage response activation in mismatch repair-deficient cells occurs due to formation of breaks in the genome after NEU damage, we investigated the formation of γH2AX foci in DLD1 cells after exposure to NEU (Figure 2E). We observed that similar to mismatch repair-proficient cells, γH2AX foci were prevalent in DLD1 cells after 1 hour of NEU damage. These results collectively suggest that activation of DNA damage cascades after exposure to the S_N_1 type-ethylating agent NEU for 2 hours is largely mismatch repair-independent.

### Activation of ATM-Chk2 checkpoint pathway precedes but is not required for activation of ATR-Chk1 response pathway

**Figure 3:**
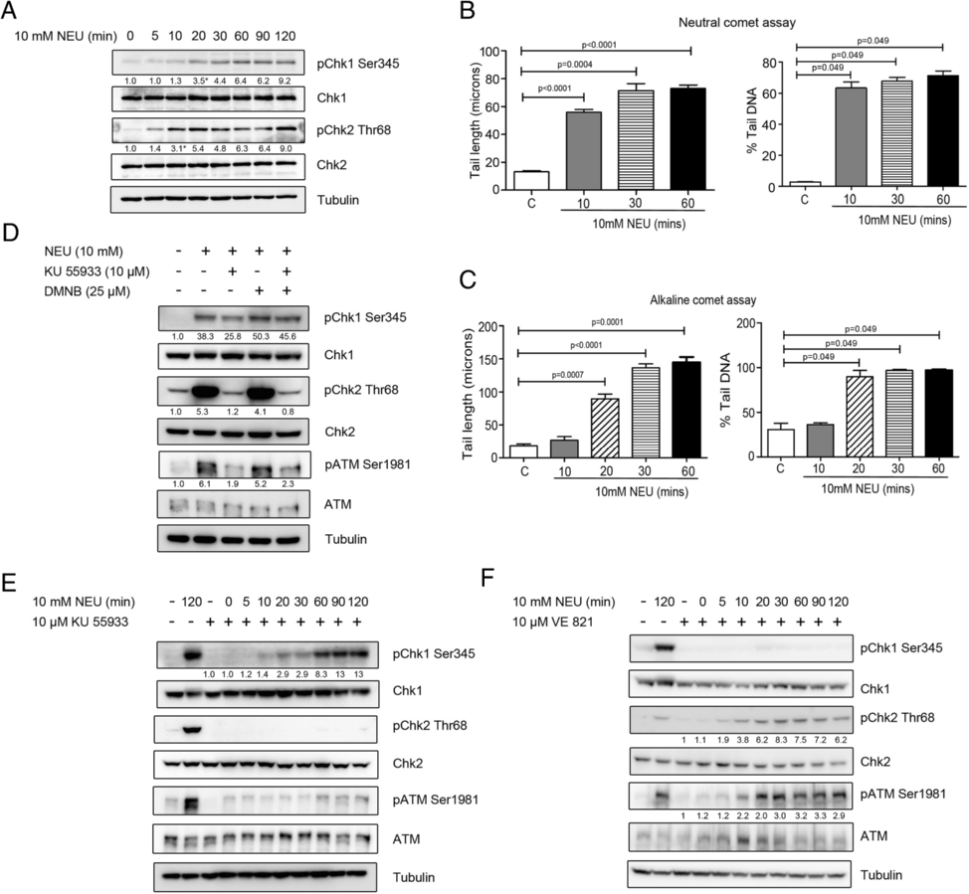
**ATM and Chk2 activation precedes but is not required for Chk1 activation. (A)** MCF7 cells were treated with 10 mM NEU for different time intervals and lysates were analysed for activation of checkpoint proteins by immunoblotting. **(B and C)** MCF 7 cells treated with 10 mM NEU at different time points were used for analysing DNA DSBs (neutral comet assay) and SSBs (alkaline comet assay) respectively. N = 150 cells (50 cells/experiment). Data shown are mean +/− standard error. The results for % DNA in tail and tail length are significantly different at p < 0.05 in Mann Whitney U test and student’s t test respectively. **(D)** MCF7 cells were treated with 10 mM NEU in presence of 10 μM KU 55933 (ATM inhibitor) or 25 μM DMNB (DNA-PK inhibitor) or a combination of both for 2 hours and lysates were analysed for activation of checkpoint proteins by immunoblotting. **(E)** MCF7 cells were treated with 10 mM NEU in presence of 10 μM KU 55933 for different time intervals and lysates were analysed for activation of checkpoint proteins by immunoblotting. **(F)** MCF7 cells were treated with 10 mM NEU in presence of 10 μM VE 821 for different time intervals and lysates were analysed for activation of checkpoint proteins by immunoblotting.

**Figure 4:**
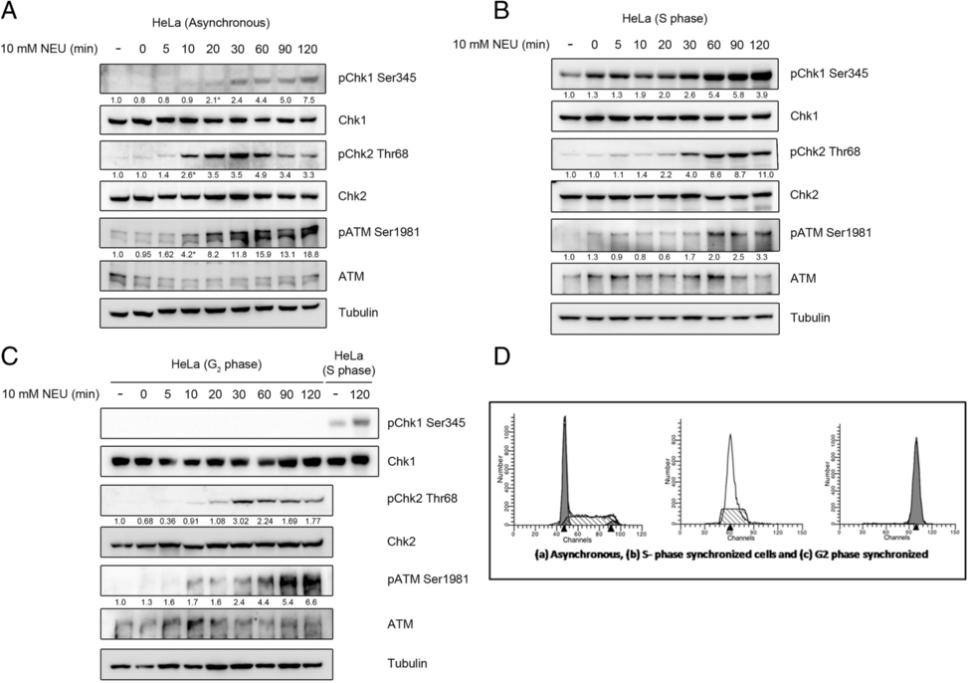
**NEU-induced activation of DNA damage response pathway is cell cycle phase dependent. (A)** HeLa cells were treated with 10 mM NEU for different time intervals and lysates were analysed for activation of checkpoint proteins by immunoblotting. **(B)** HeLa cells synchronised in S phase using double thymidine block were treated with 10 mM NEU for different time intervals and lysates were analysed for activation of checkpoint proteins by immunoblotting. **(C)** HeLa cells synchronised in G2 phase using thymidine-nocodazole block were treated with 10 mM NEU for different time intervals and lysates were analysed for activation of checkpoint proteins by immunoblotting. **(D)** Cell cycle profile of propidium iodide (PI) stained asynchronous, S phase synchronised and G2-M phase synchronised HeLa cells using Flow Cytometer.

Since we observed phosphorylation of ATM and Chk2 as well as that of Chk1 kinase after treatment of MCF7 and HeLa cells for 2 hours with NEU, we investigated the temporal sequence of activation of these two canonical DNA damage response pathways. On performing a time course assay in MCF7 and HeLa cells, we could detect phosphorylation of ATM and Chk2 10 minutes after initial exposure to NEU while Chk1 phosphorylation was detected 20 minutes after drug damage (Figures 3A and 4A). This pattern of checkpoint activation was similar to a previously reported ATM-to-ATR switch that has been shown to be involved in resection of DSBs [32]. To confirm our results regarding temporal activation of DNA damage response pathways, MCF7 cells treated with 10mM NEU for similar time points were subjected to neutral and alkaline comet assay (Figure 3B and Figure 3C). In the neutral comet assay (Figure 3B and Additional file 2: Figure S2C), tails were visible at 10 minutes post NEU damage. The comet tails in the NEU-damaged cells were significantly longer with higher percentage of DNA compared to the control cells. In the alkaline comet assay, comet tails were observed 20 minutes after NEU treatment (Figure 3C and Additional file 2: Figure S2D). There was not much tail formation in the 10 minute NEU-treated cells while the 20 minute NEU-damaged cells showed a significant increase in tail length and percentage tail DNA when compared to the untreated cells. KU 55933, an inhibitor of ATM autophosphorylation was used to investigate whether activation of upstream kinases in the DSB response pathway is essential for activation of the SSB response kinase Chk1 after DNA damage. Interestingly, though ATM and Chk2 phosphorylation were almost completely diminished after pre-treatment of MCF7 cells with KU 55933 prior to NEU treatment, unlike the findings in the previous study [32], Chk1 phosphorylation remained unhampered and was observed in 10 mM NEU damaged MCF7 cells treated with the ATM inhibitor (Figure 3D). DMNB, a DNA-PK inhibitor, was used either separately or along with KU 55933 in this experiment since members of the PIKK family of kinases show functional redundancy in ATM-deficient cells [33]. However DNA-PK inhibition did not have any significant effect on the phosphorylation profile of checkpoint proteins after NEU damage (Figure 3D). To investigate whether inhibition of DSB response pathway alters the temporal profile of Chk1 activation after treatment with NEU, a time course assay was performed in MCF7 cells pre-incubated with KU 55933. ATM and Chk2 phosphorylation was totally abolished at all time points in cells pre-treated with the ATM inhibitor before addition of NEU. Interestingly, Chk1 phosphorylation appeared at the same time point (20 minutes) after NEU damage (2.9 fold difference over control) as was observed in the absence of ATM inhibition (Figure 3E). To completely rule out cross-talk between the two canonical signalling pathways, VE 821, a potent ATP-competitive inhibitor of ATR [34] was added to cells prior to NEU treatment and a time-course assay was performed (Figure 3F). VE 821 compleletly abrogated Chk1 phosphorylation in cells damaged with NEU at all time points. However, both Chk2 and ATM phosphorylation was observed at 10 mins post NEU treatment (3.8 and 2.2 fold difference over control for pChk2 and pATM respectively). In summary, these results point towards a temporal delay in activation of the Chk1 kinase in comparison to that of ATM-Chk2 kinases after DNA damage induced by NEU, however activation of both pathways are independent of each other.

### NEU-induced activation of SSB response pathway is cell cycle phase dependent

Since the nature of DNA damage induced by alkylating agents has been proposed to be dependent on replication of DNA at the damaged site [35,36], we compared the temporal profile of checkpoint activation after NEU damage in asynchronous and phase-synchronised HeLa cell populations (Figure 4). In asynchronous HeLa cells, ATM and Chk2 were phosphorylated after 10 minutes of NEU damage while Chk1 phosphorylation was detected after 20 minutes of initial exposure to the drug (Figure 4A). Interestingly, Chk1 phosphorylation was detected as early as 0 minutes after NEU damage in S phase synchronised HeLa cells (Figure 4B). Also, these cells showed a delayed activation of ATM and Chk2 following exposure to NEU (Figure 4B). In cells synchronised in the G2-M phase, the temporal profile of ATM and Chk2 activation after NEU treatment was slightly delayed to that in asynchronous cells (30 minutes instead of 20 minutes as shown in Figure 4C. However NEU did not induce activation of Chk1 even after 2 hours of treatment to G2-M phase synchronised HeLa cells (Figure 4C). Collectively, we conclude that the profile of NEU-induced activation of ATM and Chk2 kinases was conserved across cell cycle phases while the susceptibility to activation of Chk1 kinase after DNA damage induced by NEU was highest in the S phase and least in the G2-M phase. A temporal delay in activation of ATM-Chk2 in S phase may be due to a delay in formation of DSBs during that phase.

### NEU disrupts cell polarity and induces upregulation of vimentin in MCF10A acini grown in 3D matrices

**Figure 5:**
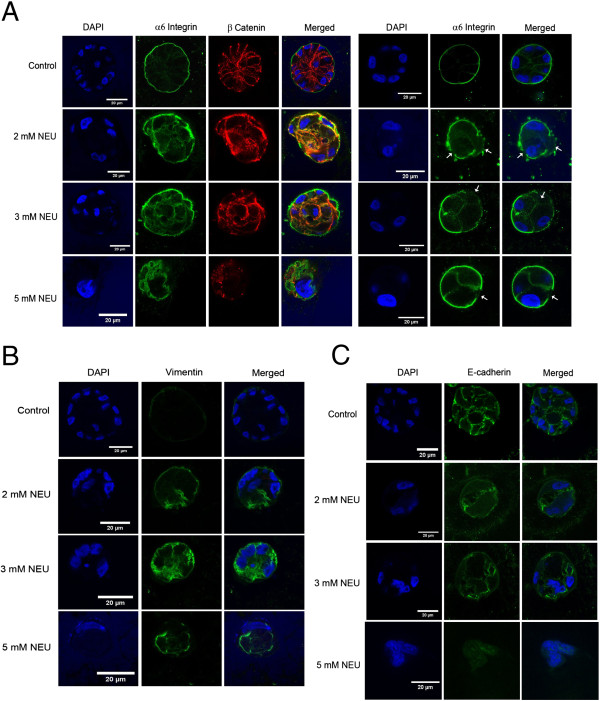
**NEU induces upregulation of vimentin and disrupts polarity in MCF10A breast acini.** MCF10A cells were grown as 3D ‘on top’ cultures in Matrigel™. 2, 3 and 5 mM NEU was administered on Day 0 and Day 2. The acini were cultured for 20 days and then immunostained for **(A)** α6-integrin (green), β-catenin (red) and DAPI (blue) to stain nuclei **(B)** Vimentin (green) a marker for EMT and **(C)** E-cadherin (green). The data is representative of 40 – 50 acini from three biologically independent experiments.

NEU was shown to disrupt polarisation in MCF10A breast epithelial cells grown as 3D ‘on top’ cultures. MCF10A epithelial cells when grown on Matrigel® differentiate to form polarised acinar structures with hollow lumen attached to the basement membrane, as shown in Figure 5A. On treatment with two doses of NEU (day 0 and 2), the polarisation appears to get disrupted, as seen by the presence of α-6 integrin on the baso-lateral and apical regions, rather than its strong basal and weak lateral localisation. Also, a few acini showed loss of integrin in certain regions ( as shown by white arrows in Figure 5A). Similar loss has been observed in cells that metastasise to the parenchyma and pleural cavity [37-39]. β-catenin was found to be disrupted and its presence was seen in the cytoplasm rather than at the cell-cell junctions (membraneous localisation). In addition to the disruption of cell polarity, an upregulation of vimentin was observed following NEU-treatment (Figure 5B). E-cadherin also showed a marginal decrease (Figure 5C) indicating a reduction of this epithelial cell marker.

## Discussion

Here, we report a novel finding that damage induced on DNA by a prototypical S_N_1 type-ethylating agent, NEU caused rapid activation of major kinases (ATM, Chk2 and Chk1) involved in the checkpoint signalling pathway as well as has the potential to cause transformation in breast acini grown as 3D cultures. The activation of the kinases is cell cycle phase dependent and is temporally controlled without any cross talk between the two parallel signalling pathways, ATM and ATR. Interestingly, mismatch repair system does not seem to play a role at the doses of NEU used in the experiments and the time of exposure of the cells to the chemical agent.

Activation of both Chk1 and Chk2, which are the two major signal relay proteins in the checkpoint signalling cascade and ATM, an apical sensor kinase, were observed in the presence of increasing dose of NEU. Activation of the above-mentioned kinases following NEU damage is indicative of lesions, both DSBs and SSBs being formed on DNA. This is interesting since within a short time interval of NEU damage, there is an immediate checkpoint response which is in contrast to earlier studies where alkylation damage forms detectable lesions following one to two cell division cycles (10). Neutral and alkaline comet assays [19,40-42] as well as phosphorylation of γH2AX on serine 139 and phosphorylation of RPA on threonine 21 confirmed the presence of breaks, both double and single stranded following 2 hours of NEU damage to cells.

**Figure 6:**
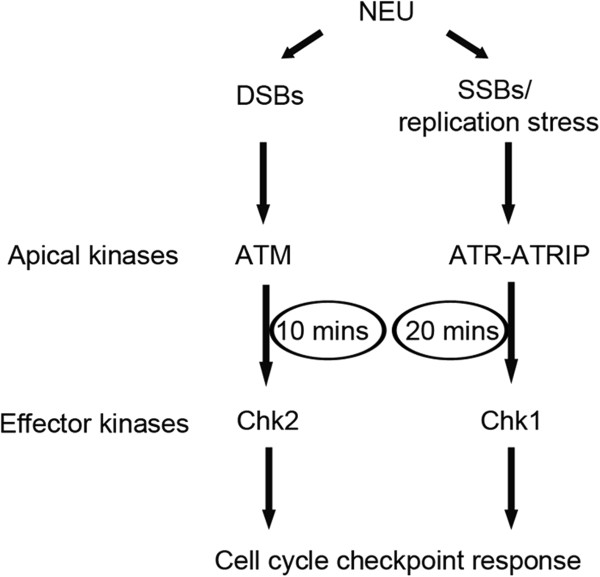
**Model depicting temporal activation of checkpoint signalling cascade following NEU damage on cells.** According to the model, ATM-Chk2 pathway gets activated at 10 minutes post NEU damage followed by activation of Chk1 signalling cascade at 20 minutes.

Activation of the DNA damage checkpoint pathway is thought to involve the independent recruitment and localisation of the ATM-Chk2 and ATR-Chk1 pathways [11-13]. Challenging this notion, recent reports have shown the existence of considerable cross talk between the two pathways [14,43]. Our data showed the activation of both ATM-Chk2 and ATR-Chk1 pathways in a temporal manner with activation of ATM-Chk2 and Chk1 kinases at 10 minutes and 20 minutes respectively after addition of NEU for 2 hours to the cells (as depicted in Figure 6). This pattern of NEU-induced checkpoint activation is similar to a previous study where IR-induced damage resulted in an ATM–to–ATR switch via single-stranded intermediates [32]. The absence of cross talk between the two signalling modules was ruled out by using ATM and ATR kinase inhibitors, KU 55933 and VE 821 respectively.

It has been previously reported that damage caused by DNA alkylating agents is recognized and repaired by the mismatch repair pathway, which includes the MutSα complex comprising of Msh2 and Msh6 proteins [10,17,29,30]. A number of downstream targets including Chk1, Chk2, p53 and CDC25A have shown to be activated in an mismatch repair-dependent manner [17,44], thereby positioning mismatch repair proteins at a level above ATR kinase. We observed activation of Chk1 and Chk2 kinases, after individual knockdown of Msh6 or Msh2 in HeLa cells post 10 mM NEU damage. Interestingly, knock down of Msh2 also led to a decrease in Msh6 expression in the cells and *vice versa*. This is corroborative with a previous study where the expression of both proteins was required to maintain a steady-state regulation of the mismatch repair system [45]. Our observation was further confirmed when we observed phosphorylation of Chk1 and Chk2 following 2 hours of NEU induced damage in mismatch repair-deficient cell lines, namely HCT116 and DLD1. Overall, the checkpoint activation profile in mismatch repair deficient cell lines post NEU damage was found to be similar to that of mismatch repair proficient cells.

In order to test the hypothesis that cells in different phases of the cell cycle could respond differently to the DNA damaging agent, cells were synchronised at S and G2/M phases. S-phase synchronised cells showed activation of Chk1 as soon as they were released from the block, while phosphorylation of Chk2 was observed from 20 minutes. The immediate activation of Chk1 may be speculated to be due to sensitivity of the cells to NEU damage during the replication cycle. There is a higher propensity of single-stranded regions being exposed during this cell cycle phase and therefore immediate activation of Chk1. ATM as well as Chk2 activation was delayed since activation of this pathway requires DSBs or changes in chromatin structure [46]. Interestingly, the checkpoint activation profile was observed to be different during G2/M synchrony where a complete abrogation of Chk1 was observed while ATM and Chk2 activation was observed at 30 minutes post release from G2/M block in NEU drug. This may be speculated to be due to a higher incidence of sister chromatid exchange (SCE) in cells treated with NEU as has been previously observed by Kaina and his colleagues [47] and therefore propensity for increased DSBs to occur during that time. Another reasoning could be the presence of abasic sites formed following the removal of the NEU adduct formed on DNA, giving rise to gaps which may be recognised as DSBs which then leads to the activation of ATM-Chk2 kinases as has been observed in our data.

This study has shed light on some of the players of the DNA damage surveillance pathway that are activated when a prototypical S_N_1 type-ethylating agent, NEU, causes insult to DNA. There is a good number of studies on methylating agents and their possible mechanism of action in cells as well as their effect on some of the cellular pathways such as DNA repair. Most research articles have addressed O^6^meG and repair by mismatch repair, but literature addressing O^6^EtG lesions and its repair is lacking. Further investigations are necessary to understand the DNA repair pathways that may be involved in repairing the lesions induced by incorporation of an ethyl group to DNA as well as any other sensor protein complex that may first detect the mis-incorporation and then signal to the apical checkpoint kinases. Although it has been reported that DNA alkylating agents do not directly form DSBs, but are formed after processing of lesions induced by the alkylating agent, our study provides evidence that on addition of NEU, the ATM and Chk2 pathway is activated, as early as 10 minutes. This is in contrast to earlier published work where γH2AX foci formation was detected after 24 hours [46]. Therefore, it will be interesting to investigate how NEU alkylation damage is able to convert to a DSB lesion that is capable of activating the DSB response pathway within a short interval of time.

NEU is known to cause point mutations which ultimately lead to the formation of mammary tumours in rat models. NEU has been shown to induce neoplastic transformation *in vitro* of rat mammary epithelial cell [48]. *In vitro* studies have also shown NEU to act as an active rat mammary gland genotoxic carcinogen [4,49]. During the process of neoplastic transformation, one of the earliest stages of invasion is epithelial to mesenchymal transition (EMT) wherein the epithelial cells acquire mesenchymal characteristic so as to invade the surrounding extracellular matrix and migrate towards distant organs [50,51]. EMT is characterised by loss of polarity of the epithelial cells, appearance of mesenchymal markers (upregulation of vimentin, fibronectin, N-cadherin) and down regulation of the epithelial markers (E-cadherin, occludins, cytokeratin 19, claudins) [52,53]. During EMT, β-catenin which is membranous has been found to relocalise in the cytoplasm and/or nucleus [54]. Immortalised breast epithelial cells (MCF10A) when treated with NEU showed upregulation of vimentin. There was marginal loss of E-cadherin following treatment, and complete loss at 5 mM NEU treatment. NEU treatment at all doses also led to disruption of polarity of cells in the acini, overall giving rise to an EMT-like phenotype. Thus, it may be speculated that NEU may play a role in causing transformation in breast acini grown as 3D cultures.

## Conclusions

In conclusion, our study reports two novel findings. First, NEU causes DNA lesions within 2 hours of administration that causes the activation of checkpoint signalling kinases, Chk1 and Chk2 in a temporal manner. This activation does not depend upon the mismatch repair complex and is cell cycle phase-dependent. The second finding is that NEU can cause disruption of polarity in cells forming the breast acini grown in 3D as well as upregulate vimentin, thereby leading to transformation *in vitro*. Therefore, NEU can potentially be used as an agent to induce such a phenotype. This strategy will not only permit the study of novel genes that are required for normal mammary development but also shed light on genes that get disrupted in breast cancer.

## Abbreviations

NEU: N-nitroso-N-ethylurea; SSBs: Single strand breaks; DSBs: Double strand breaks; EMT: Epithelial to Mesenchymal transition; MMR: Mismatch repair; O6EtG: *O*^*6*^-ethylguanine; DDR: DNA damage response; ATM: Ataxia-telangiectasia mutated; ATR: ATM and Rad3-related; PIKK: Phosphoinositide 3-kinase related kinase; RT: Room temperature.

## Competing interests

The authors declare that they have no competing interests.

## Authors’ contributions

All the authors contributed to the design of the project. Monolayer culture experiments were performed by SB, SS and PT, while three dimensional culture experiments were performed by LAV. ML, SB, SS and LAV wrote the manuscript. All authors read and approved the final manuscript.

## Pre-publication history

The pre-publication history for this paper can be accessed here:

http://www.biomedcentral.com/1471-2407/14/287/prepub

## Supplementary Material

Additional file 1: Figure S1 Checkpoint activation in MCF7 and HeLa cells post NEU damage. **(A)** and **(B)** MCF7 and HeLa cells respectively were treated with increasing NEU concentrations ranging from 0.2 mM to 18 mM for 2 hours. Percent viability was determined for each NEU dose by normalising corresponding absorbance at 570 nm with respect to that of untreated cells. **(C)** HeLa cells were treated with 0, 2, 6 and 10 mM NEU for 2 hours and lysates were analysed for activation of checkpoint proteins by immunoblotting. **(D)** HeLa cells were treated with 0, 0.3, 0.6, 1.2 and 1.8 mM NEU for 2 hours and lysates were analysed for activation of checkpoint proteins by immunoblotting. **(E)** MCF7 cells were treated with 10 mM NEU for 1 hour, fixed and analysed for γH2AX foci formation by immunostaining. DMSO was used as negative control and 200 ng/ml neocarzinostatin (NCS), an IR mimetic drug, was used as positive control. Scale bar: 20 μM. Click here for file

Additional file 2: Figure S2 NEU induced formation of DSBs and SSBs in MCF7 cells. DNA damage in NEU treated MCF7 cells were measured using comet assay. Cells treated with 10 mM NEU for two hours were subjected to **(A)** neutral comet assay and **(B)** alkaline comet assay. **(A and B)** Representative images of ethidium bromide stained control cells, showing intact super coiled DNA (left) and treated cells showing damaged DNA migrating out of the cell (right). **(C and D)** Cells treated with 10 mM NEU at different time points were subjected to neutral comet assay and alkaline comet assay. Representative images of ethidium bromide stained control cells showing intact super coiled DNA and treated cells at **(C)** 10, 30 and 60 minutes for neutral comet and **(D)** 10, 20, 30 and 60 minutes for alkaline comet showing damaged DNA migrating out of the cell.Scale bar: 50 μM. Click here for file
