# Supplementary figures and images for: N-nitroso-N-ethylurea activates DNA damage surveillance pathways and induces transformation in mammalian cells
Source: BMC Cancer. 2014 Apr 24;14:287. doi: 10.1186/1471-2407-14-287 (PMC4021545; doi:10.1186/1471-2407-14-287)

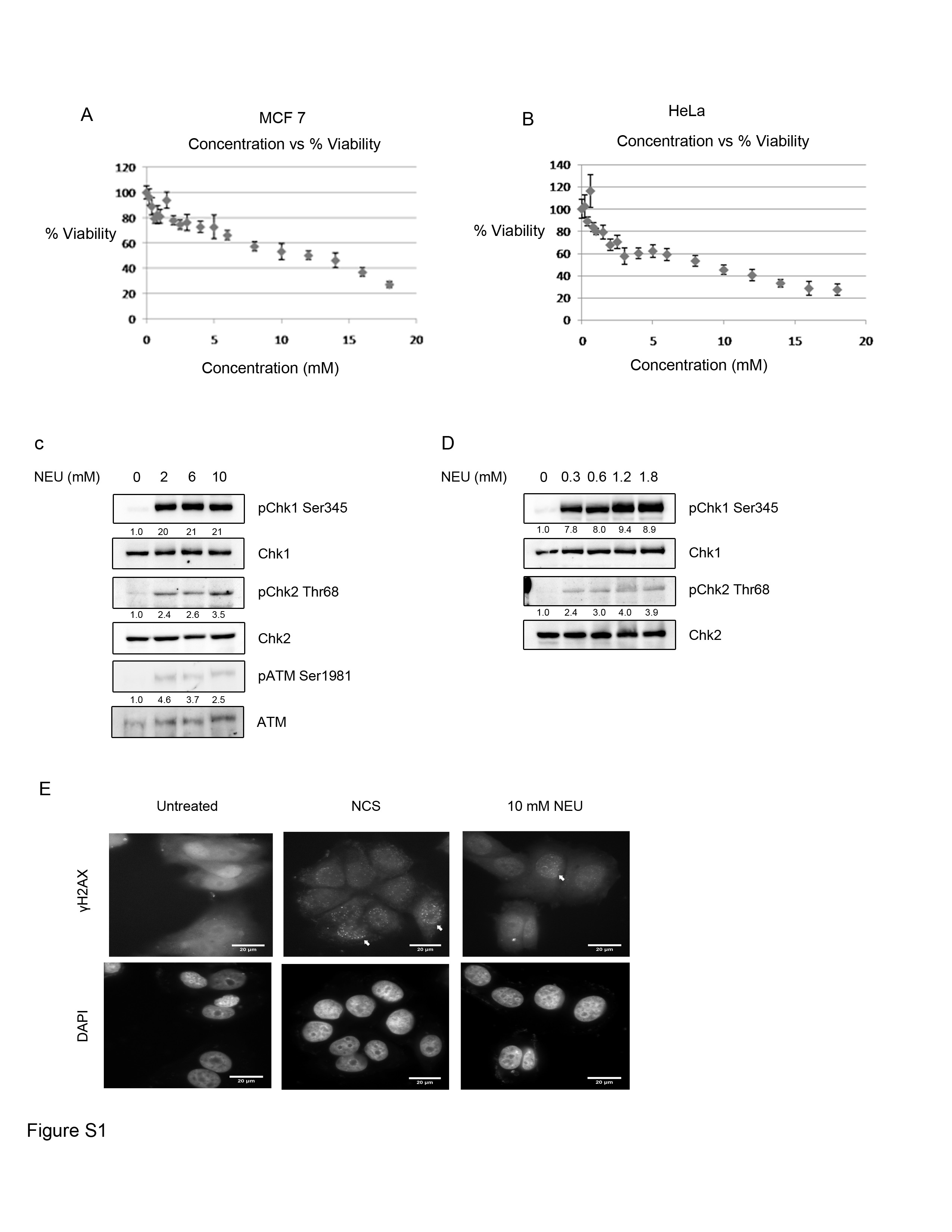

Supplement: Additional file 1: Figure S1 — Checkpoint activation in MCF7 and HeLa cells post NEU damage. (A) and (B) MCF7 and HeLa cells respectively were treated with increasing NEU concentrations ranging from 0.2 mM to 18 mM for 2 hours. Percent viability was determined for each NEU dose by normalising corresponding absorbance at 570 nm with respect to that of untreated cells. (C) HeLa cells were treated with 0, 2, 6 and 10 mM NEU for 2 hours and lysates were analysed for activation of checkpoint proteins by immunoblotting. (D) HeLa cells were treated with 0, 0.3, 0.6, 1.2 and 1.8 mM NEU for 2 hours and lysates were analysed for activation of checkpoint proteins by immunoblotting. (E) MCF7 cells were treated with 10 mM NEU for 1 hour, fixed and analysed for γH2AX foci formation by immunostaining. DMSO was used as negative control and 200 ng/ml neocarzinostatin (NCS), an IR mimetic drug, was used as positive control. Scale bar: 20 μM. [file 1471-2407-14-287-S1.jpeg]

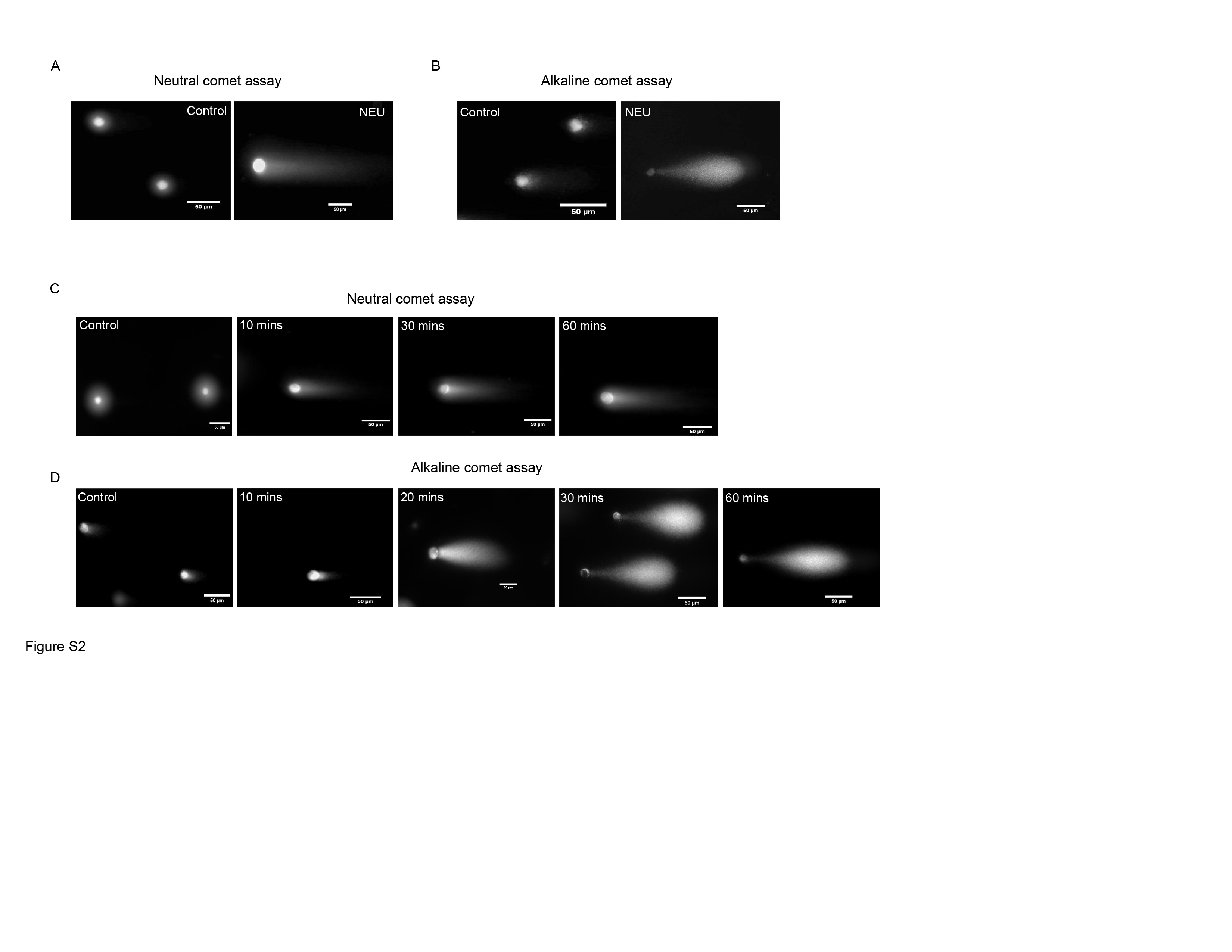

Supplement: Additional file 2: Figure S2 — NEU induced formation of DSBs and SSBs in MCF7 cells. DNA damage in NEU treated MCF7 cells were measured using comet assay. Cells treated with 10 mM NEU for two hours were subjected to (A) neutral comet assay and (B) alkaline comet assay. (A and B) Representative images of ethidium bromide stained control cells, showing intact super coiled DNA (left) and treated cells showing damaged DNA migrating out of the cell (right). (C and D) Cells treated with 10 mM NEU at different time points were subjected to neutral comet assay and alkaline comet assay. Representative images of ethidium bromide stained control cells showing intact super coiled DNA and treated cells at (C) 10, 30 and 60 minutes for neutral comet and (D) 10, 20, 30 and 60 minutes for alkaline comet showing damaged DNA migrating out of the cell.Scale bar: 50 μM. [file 1471-2407-14-287-S2.jpeg]
